# A bioinformatics analysis of the correlation between hepatic ischemia–reperfusion injury and postoperative cognitive dysfunction

**DOI:** 10.3389/fgene.2025.1607446

**Published:** 2025-07-24

**Authors:** Qing Hu, Haijie Liu, Yunfei Bao, Zhihao Feng, Hongbo Zhang, Jianling Li

**Affiliations:** ^1^ The Affiliated Hospital of Chengde Medical University, Hebei Province Key Laboratory of Pan-Vascular Diseases Chengde, Hebei, China; ^2^ Chengde Medical University, Chengde, China

**Keywords:** liver ischemia–reperfusion injury, postoperative cognitive dysfunction, bioinformatics analysis, drug–gene interaction prediction, miRNA prediction

## Abstract

**Background:**

Postoperative cognitive dysfunction (POCD) is a common postoperative complication that is prevalent in elderly people. An increasing number of elderly patients are undergoing surgery. As a result, the number of patients presenting with POCD is increasing. Previous studies have demonstrated that hepatic ischemia–reperfusion injury (HIRI) in mice is associated with postoperative cognitive impairment. Therefore, this study investigated the relationship between POCD and HIRI using bioinformatics research methods.

**Methods:**

The Gene Expression Omnibus (GEO) database GSE202565 and GeneCards data were selected for correlation analysis using bioinformatics analysis methods. The GSE112713 dataset from the GEO database was chosen for preliminary validation of the screened hub genes.

**Results:**

We analyzed the dataset GSE202565 for differences in gene expression before and after hepatic post-ischemic reperfusion and obtained a total of 53 genes by identifying POCD-related genes. We also screened these 53 genes again and obtained 10 hub genes, which were analyzed and used for correlation prediction. Finally, these 10 hub genes were partially and preliminarily validated using the dataset GSE112713.

**Conclusion:**

HIRI is closely related to POCD, and reducing the occurrence of HIRI may become one of the ways to avoid or improve postoperative cognitive impairment in the future.

## 1 Introduction

Postoperative cognitive dysfunction (POCD) refers to a neurocognitive decline after anesthesia and surgery, which constitutes a complication of the central nervous system (CNS) (Rundshagen, 2014). POCD is clinically characterized by a decrease in cognitive function (learning, memory, thinking, and paying attention) for several days to weeks after surgery, and some patients with POCD may even develop dementia. POCD prolongs patients’ hospital stay, increases medical costs, and ultimately imposes serious burdens on patients’ families and society (Gong et al., 2018; Berger et al., 2015). POCD is a common postoperative complication that is prevalent among elderly people. With the advancement of medical technology and the aging population in China, an increasing number of elderly patients are undergoing surgery. As a result, the number of patients presenting with POCD is also increasing, a phenomenon that puts enormous economic pressure on the country and their families (Qu et al., 2021).

Liver transplantation is now widely recognized as the only therapeutic option for end-stage liver disease, acute fulminant liver failure, hepatocellular carcinoma, hilar cholangiocarcinoma, and multiple metabolic disorders (Jadlowiec and Taner, 2016). The number of patients undergoing liver transplantation is also increasing; however, liver transplantation is inevitably accompanied by hepatic ischemia–reperfusion injury (HIRI). HIRI is a complex pathophysiological process involving multiple factors that worsen liver damage, dysfunction, and structural destruction after insufficient or interrupted blood flow to the liver and the subsequent restoration of blood flow (Klune and Tsung, 2010; Jaeschke, 2003).

**FIGURE 1:**
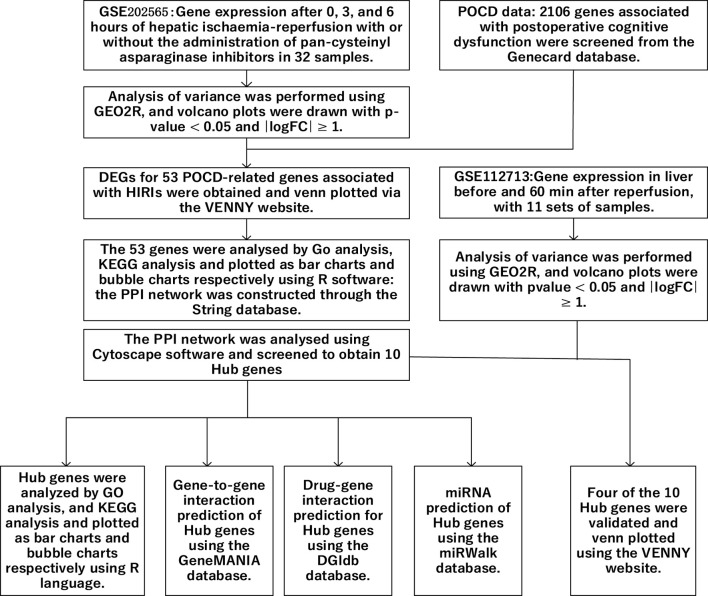
Technology roadmap: the overall design thinking for this study.

In noncardiac surgery, the frequency of POCD in patients over 60 years of age is approximately 20% (Monk et al., 2008). Some patients are at risk for neurologic complications after undergoing liver transplantation, including embolic stroke, cerebral hemorrhage, and CNS infections, which can lead to varying degrees of cognitive dysfunction (Campagna et al., 2010). The development of postoperative cognitive dysfunction in liver transplant patients may be related to the development of hepatic ischemia–reperfusion injury. Wu et al. (2019) demonstrated that short-term cognitive dysfunction occurs in a mouse model of hepatic ischemia–reperfusion injury. However, there are few studies on the exact mechanism of HIRI-induced POCD. Therefore, in this study, the relationship between POCD and HIRI was investigated using bioinformatics methods (Figure 1), providing a reference for future research on the exact mechanisms linking the two, with the aim of improving or preventing the occurrence of POCD.

## 2 Results

### 2.1 Differential analysis of HIRI-related genes and mapping of volcanoes

**FIGURE 2:**
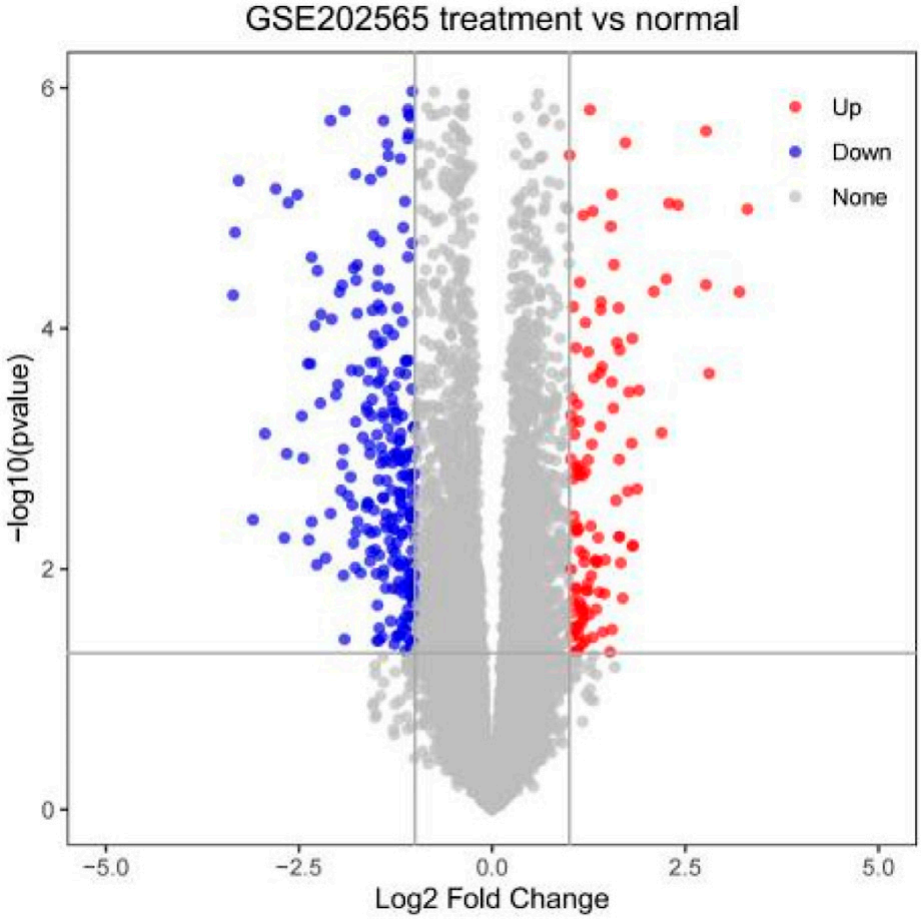
Volcano chart mapped using 259 HIRI-related DEGs.

We screened 259 relevant differentially expressed genes (DEGs) for HIRI from the dataset GSE202565 using GEO2R, including 67 upregulated and 192 downregulated genes, and mapped them in a volcano chart (Figure 2).

### 2.2 Identification of POCD-related genes and mapping of Venn

**FIGURE 3:**
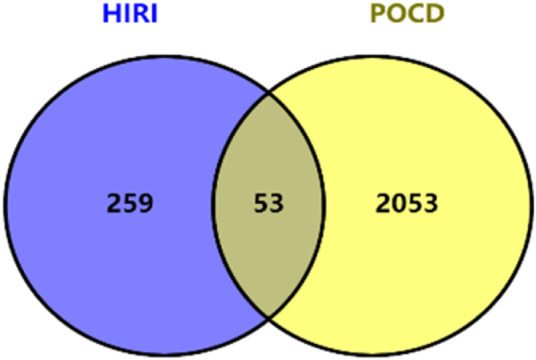
Venn chart of HIRI DEGs with “POCD-related” genes.

After intersecting 2,053 POCD-related genes with 259 HIRI-related DEGs, 53 DEGs of POCD-related genes were obtained with HIRI-related amounts (Table 1). Venn diagrams were plotted (Figure 3). It should be noted that in Table 1, all extremely small *p*-values are expressed as *p* < 0.001 instead of the actual exact *p*-values.

**TABLE 1:** Symbols of DEGs for 53 POCD-related genes, including the corresponding *p*-value, logFC, and POCD-related genes.

Symbol	*p*-value	logFC	Symbol	*p*-value	logFC
IL-6	<0.001	4.911963	CCL3	0.00138	2.26188
TNF	<0.001	3.197148	CACNA1H	0.00543	1.569451
IL-1B	<0.001	4.042976	FGF7	<0.001	1.907473
CXCL8	0.002	3.352203	CD8A	0.0202	1.41055
CCL2	0.0115	2.462572	KLRK1	0.00249	1.432327
PTGS2	<0.001	3.226491	CD69	0.0185	1.924937
MALAT1	<0.001	3.714757	LIF	0.048	2.335665
LINC02605	0.0195	2.658483	CCL4	0.00112	2.336597
SELE	0.0414	1.527353	TNFAIP3	0.0397	1.624781
GSTM1	0.0468	3.095218	BCL2A1	0.00737	1.995958
LPL	<0.001	1.835197	F2RL2	0.0354	1.41437
CCR5	0.00186	1.338599	TNFAIP8	0.0173	1.587145
PLAU	0.00266	2.215611	CYP17A1	<0.001	−1.4054429
PTX3	0.0209	2.444272	FOXP3	<0.001	−1.7752286
CXCL10	<0.001	3.632874	CYP2B6	0.00172999999	−2.7678
CCL5	0.00722	1.474243	NQO1	0.00457	−1.6504174
BMP2	0.0377	1.615078	SAA2	0.00711999	−1.547296
NOD2	0.0379	1.602128	SAA1	0.00621999	−1.3962247
BCYRN1	<0.001	5.89061	VWF	0.00905000	−1.0415559
CXCL9	0.00697	1.59966	A2M	0.02100000	−1.0197812
IL-7R	<0.001	1.771307	JPH1	0.02309999	−1.1267352
NTF3	<0.001	1.520565			

### 2.3 GO analysis of HIRI and POCD-related genes

**FIGURE 4:**
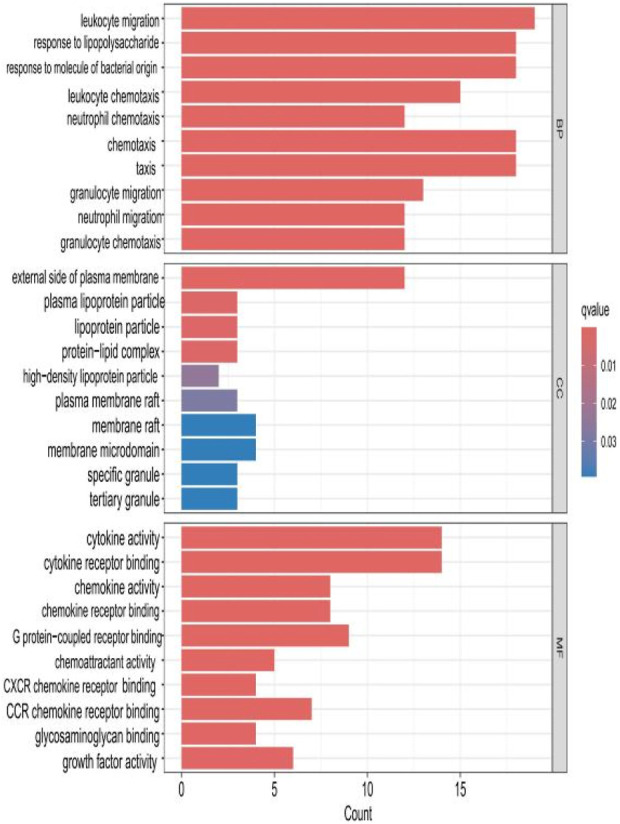
Bar chart plotted against the results of GO analysis for the 53 genes in Table 1.

**FIGURE 5:**
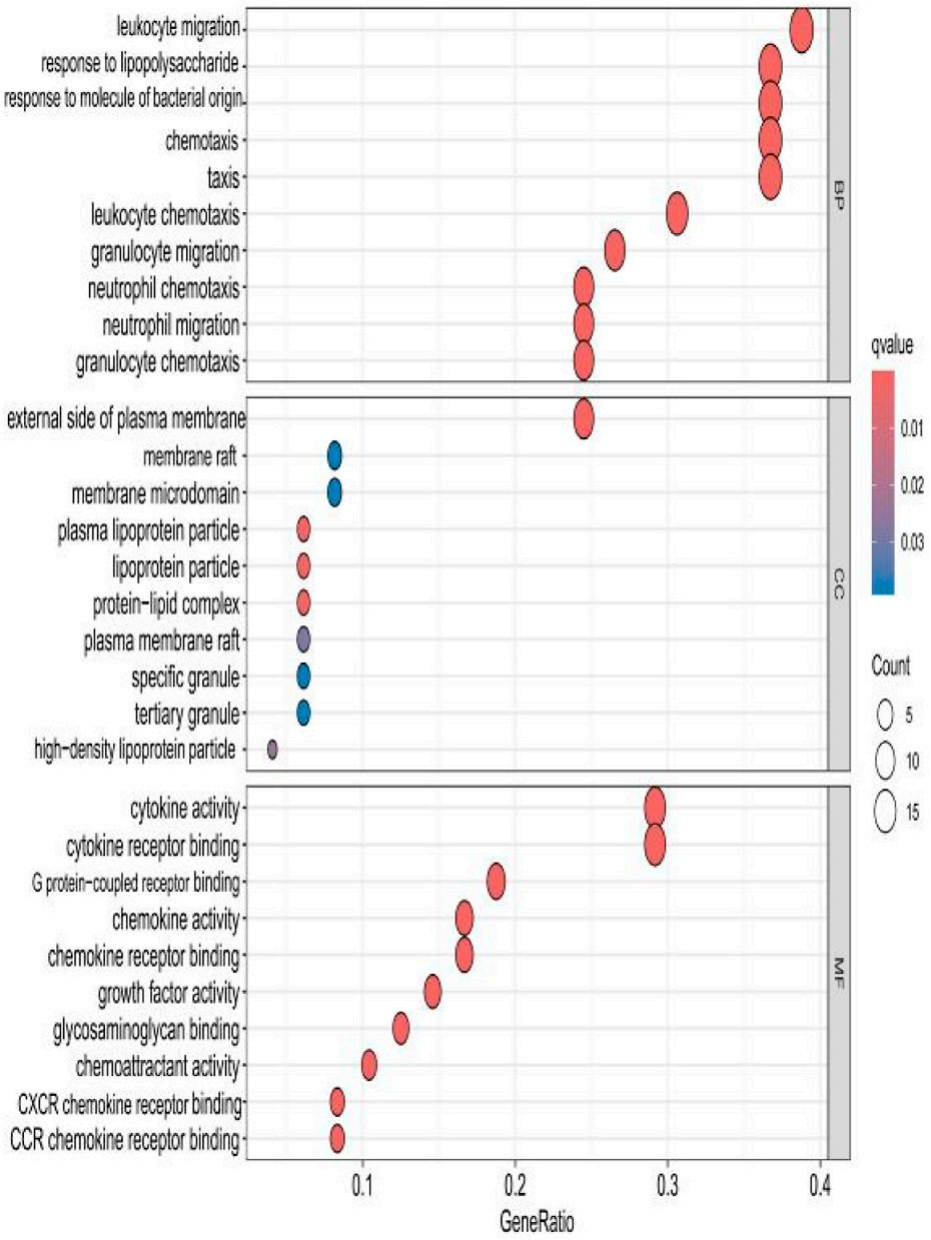
Bubble chart plotted against the results of GO analysis for the 53 genes in Table 1.

The top 10 results with the smallest *p*-values were selected from the three categories of biological process (BP), cellular component (CC), and molecular function (MF) in the Gene Ontology (GO) analysis and are plotted as bar charts (Figure 4) and bubble charts (Figure 5). From the figures, we can observe that in the BP category, the main enrichment is related to “leukocyte migration.” CC is mainly enriched in the “external side of plasma” and “endoplasmic reticulum lumen.” MF is primarily enriched in “cytokine activity” and “cytokine receptor binding.” Previous studies have found that the above processes are all interconnected through an “inflammation–neurotoxicity axis”—leukocyte migration mediates the transmission of peripheral inflammation to the brain, plasma membrane molecules (adhesion molecules and receptors) serve as key mediators, and cytokine–receptor binding acts as a core signaling hub, ultimately leading to neuroinflammation, synaptic damage, and cognitive decline.

### 2.4 KEGG analysis of HIRI and POCD-related genes

**FIGURE 6:**
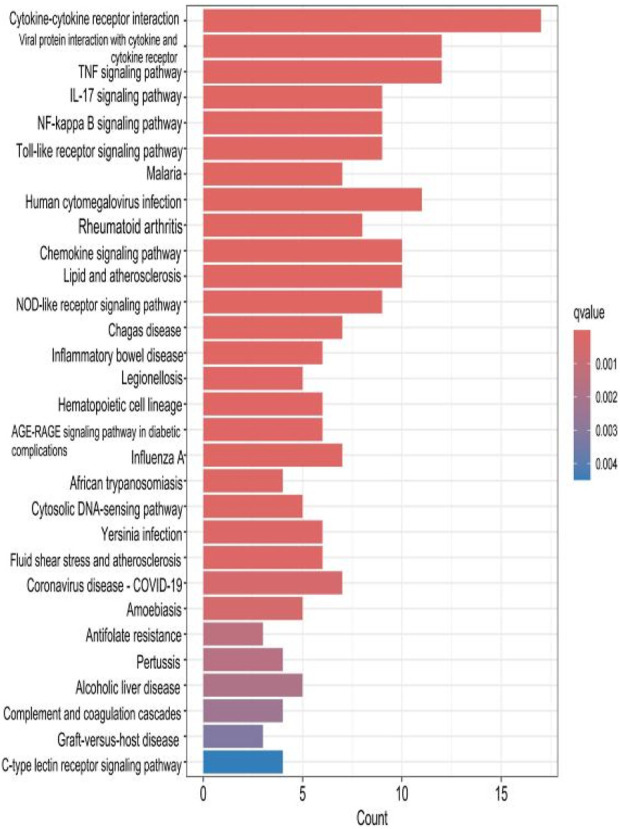
Bar chart plotted against the results of KEGG analysis for the 53 genes in Table 1.

**FIGURE 7:**
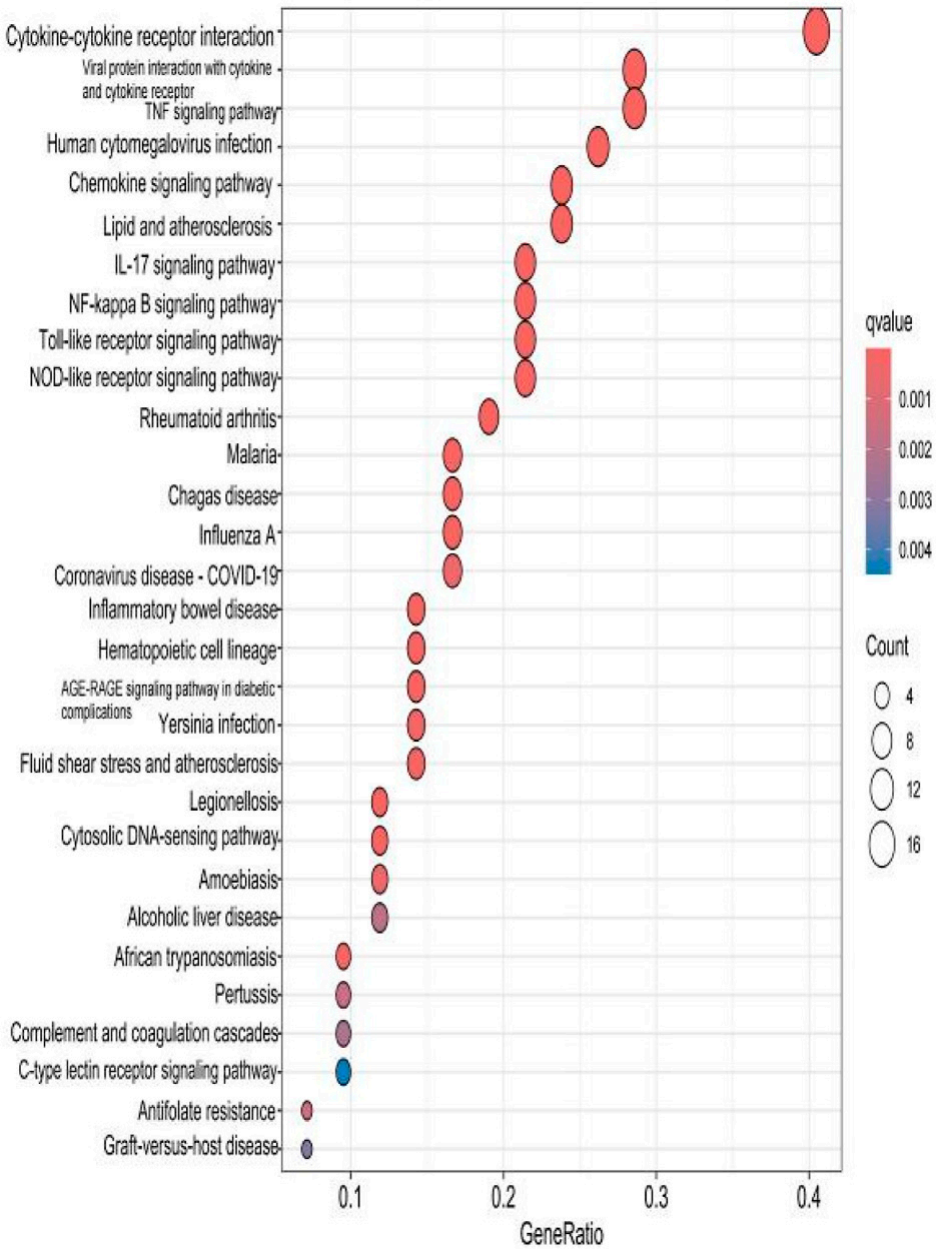
Bubble chart plotted against the results of KEGG analysis for the 53 genes in Table 1.

The top 30 results with the smallest *p*-values in the Kyoto Encyclopedia of Genes and Genomes (KEGG) analysis are plotted as bar charts (Figure 6) and bubble charts (Figure 7). From the graphs, we can observe that the analyzed results are mainly enriched in six areas, including “cytokine–cytokine receptor” and “viral protein interaction.”

### 2.5 Construction of the PPI network of HIRI- and POCD-related genes

**FIGURE 8:**
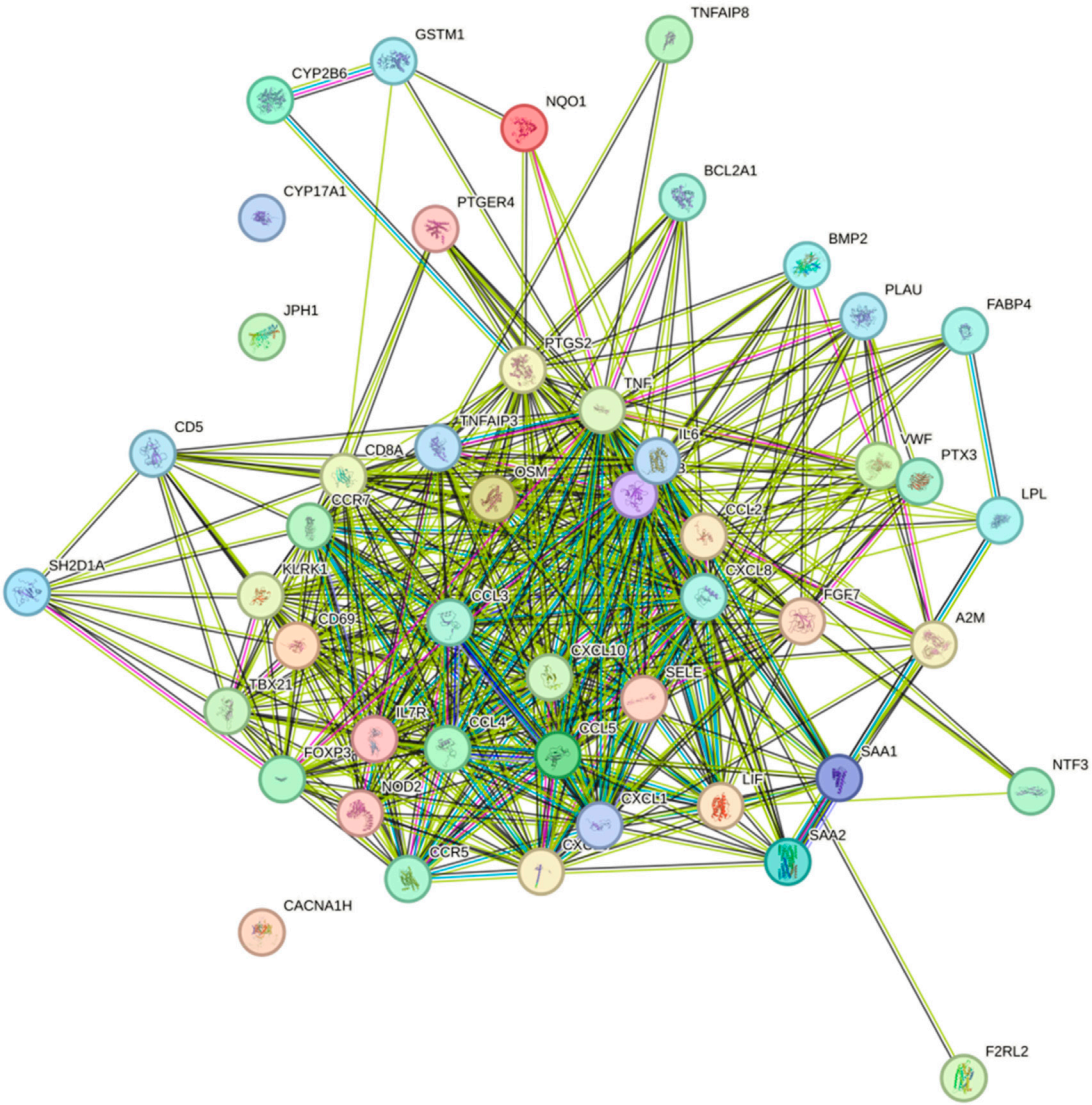
Construction of the PPI networks for the 53 genes in Table 1.

The construction of the protein–protein interaction (PPI) networks for the 53 genes in Table 1 (Figure 8) showed that 50 of the 53 genes were associated with each other. Among them, the “CYP17A1,” “JPH1,” and “CACNA1H” genes were not associated with other genes.

### 2.6 Cytoscape software analysis of PPI networks to screen for hub genes

**FIGURE 9:**
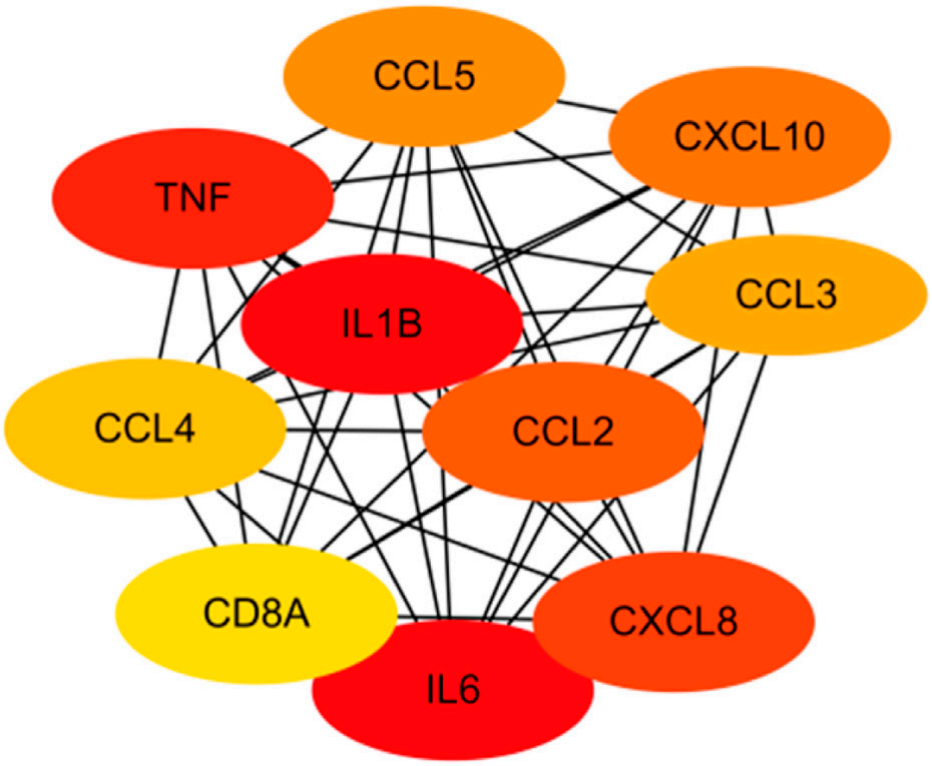
The analysis of the 53 genes in Table 1 using the MCC algorithm via the CytoHubba function in Cytoscape software yielded hub genes.

The CytoHubba application was used to analyze the constructed PPI network in Cytoscape software (Figure 9). The 53 genes were scored using the MCC algorithm, and we chose 10 genes with the highest scores as the hub genes (Table 2), namely, “chemokine C–C motif ligand 2 (*CCL2*),” “*IL-1B*,” “*CXCL10*,” “*CXCL8*,” “*CCL4*,” “*TNF*,” “*IL-6*,” “*CCL3*,” “*CCL5*,” and “*CD8A*.”

**TABLE 2:** The 53 genes in Table 1 were scored using the MCC algorithm via the CytoHubba function in Cytoscape software.

Rank	Name	Score
1	IL-1B	444821169001104
1	IL-6	444821169001104
3	TNF	444821169001086
4	CXCL8	444821165371441
5	CCL2	444821165002920
6	CXCL10	444821164220160
7	CCL5	444821163857280
8	CCL3	444821003832240
9	CCL4	444821003827200
10	CD8A	444796099817042

### 2.7 Hub gene GO analysis

**FIGURE 10:**
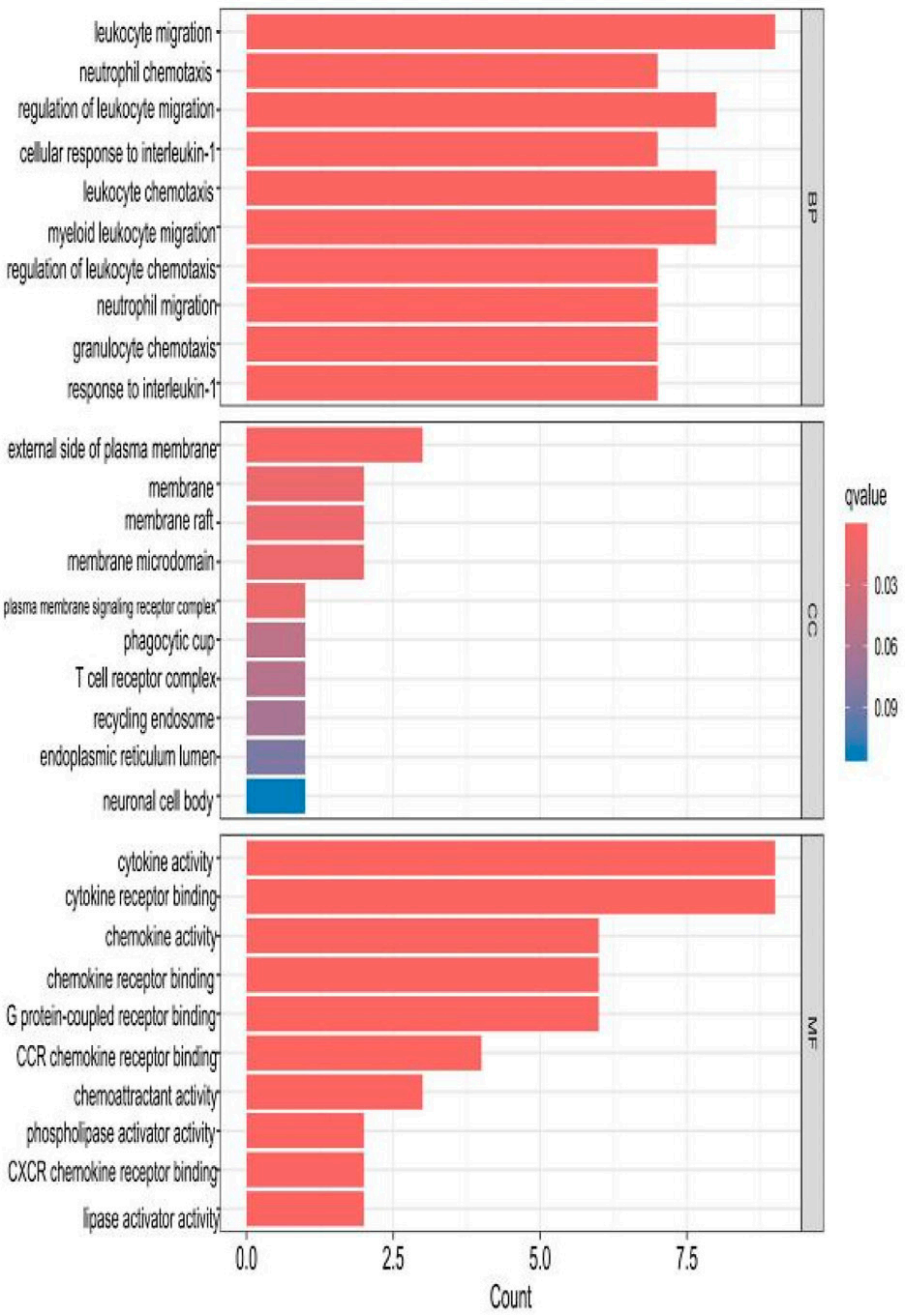
Bar chart plotted against the results of GO analysis for the hub gene.

**FIGURE 11:**
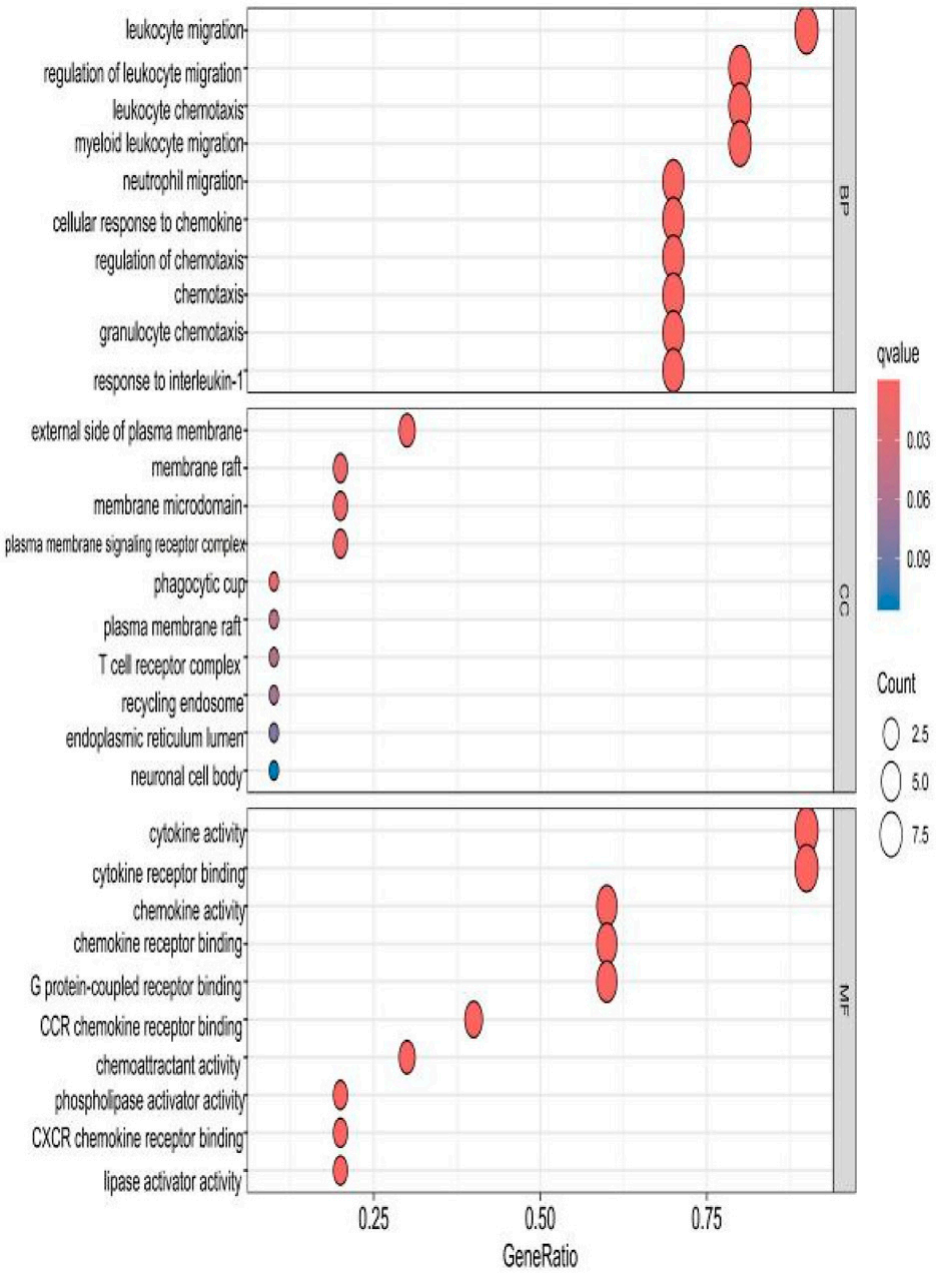
Bubble chart plotted against the results of GO analysis for the hub gene.

The top 10 results with the smallest *p*-values were selected from the three categories of BP, CC, and MF in the GO analysis and are plotted as bar charts (Figure 10) and bubble charts (Figure 11). From the graphs, it can be found that in BP, the main enrichment involves four aspects, including “leukocyte migration.” CC is mainly enriched in four aspects, including the “external side of plasma.” MF was enriched primarily in four areas, including growth factor receptor binding, cytokine activity, and cytokine receptor binding.

### 2.8 KEGG analysis of the hub gene

**FIGURE 12:**
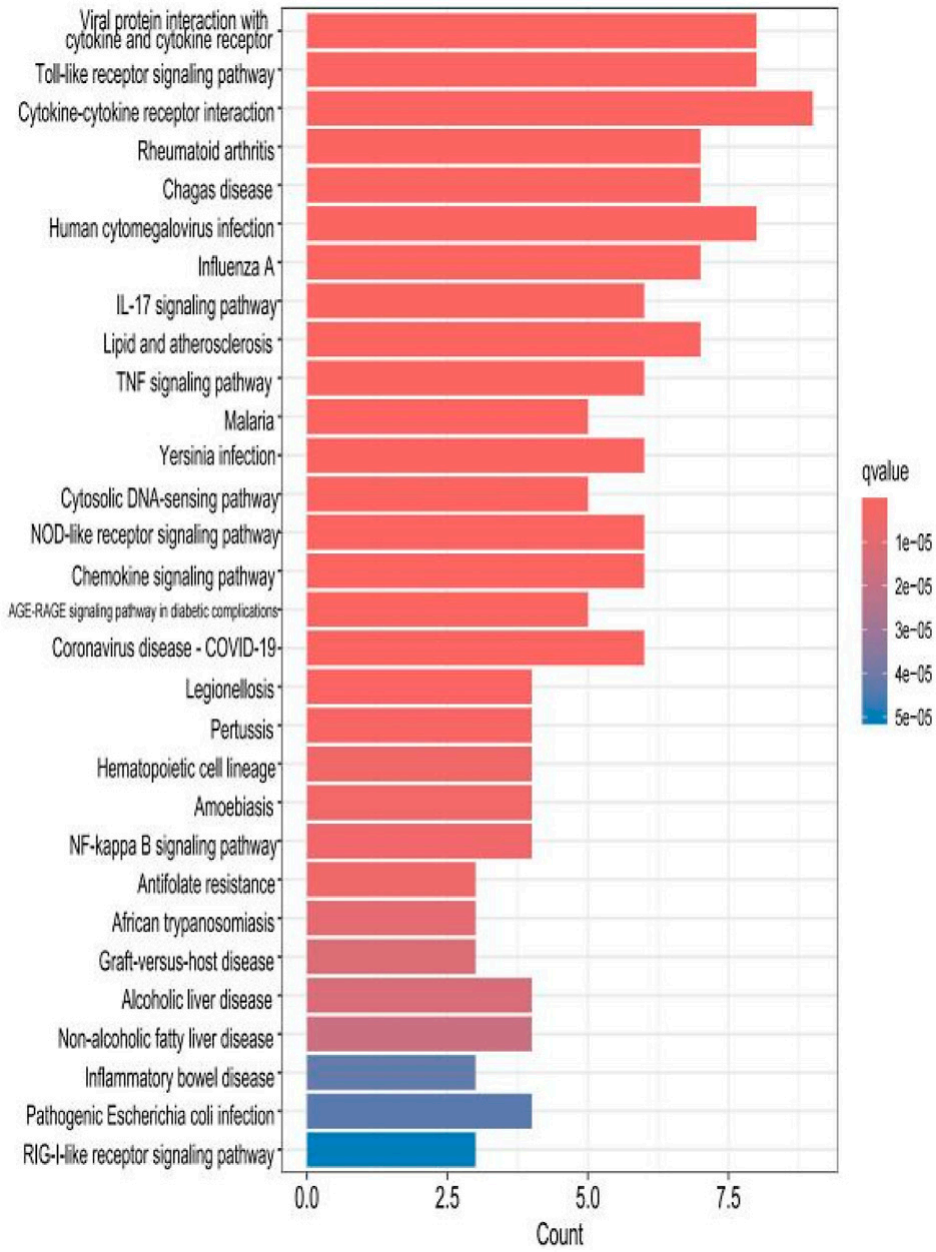
Bar chart plotted against the results of KEGG analysis for the hub gene.

**FIGURE 13:**
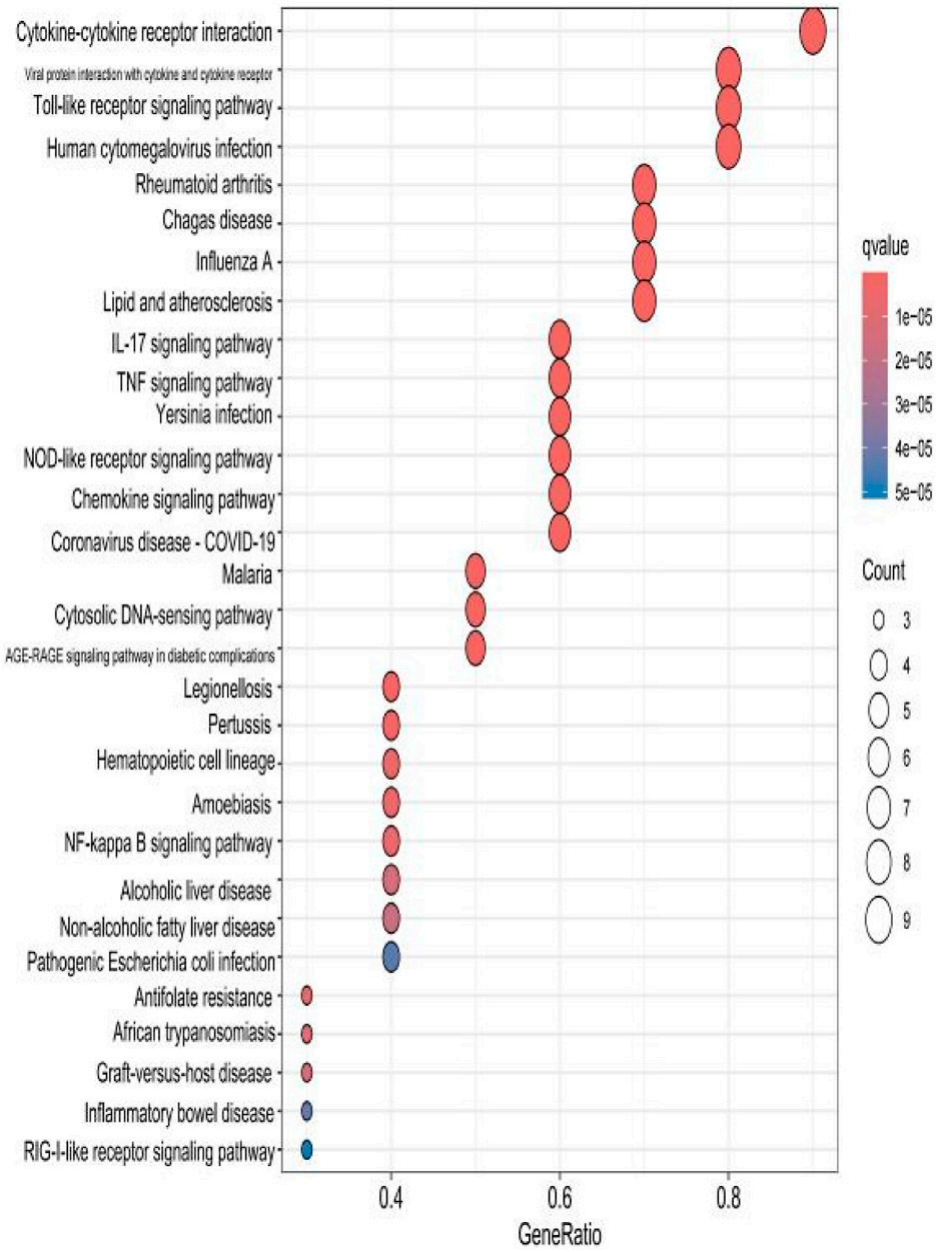
Bubble chart plotted against the results of KEGG analysis for the hub gene.

The results of the KEGG analysis with the top 30 *p*-values were selected and are plotted as a bar graph (Figure 12) and a bubble graph (Figure 13). From the graphs, it can be observed that there are four main areas of enrichment, including “cytokine–cytokine receptor”.

### 2.9 Reciprocal prediction between genes

**FIGURE 14:**
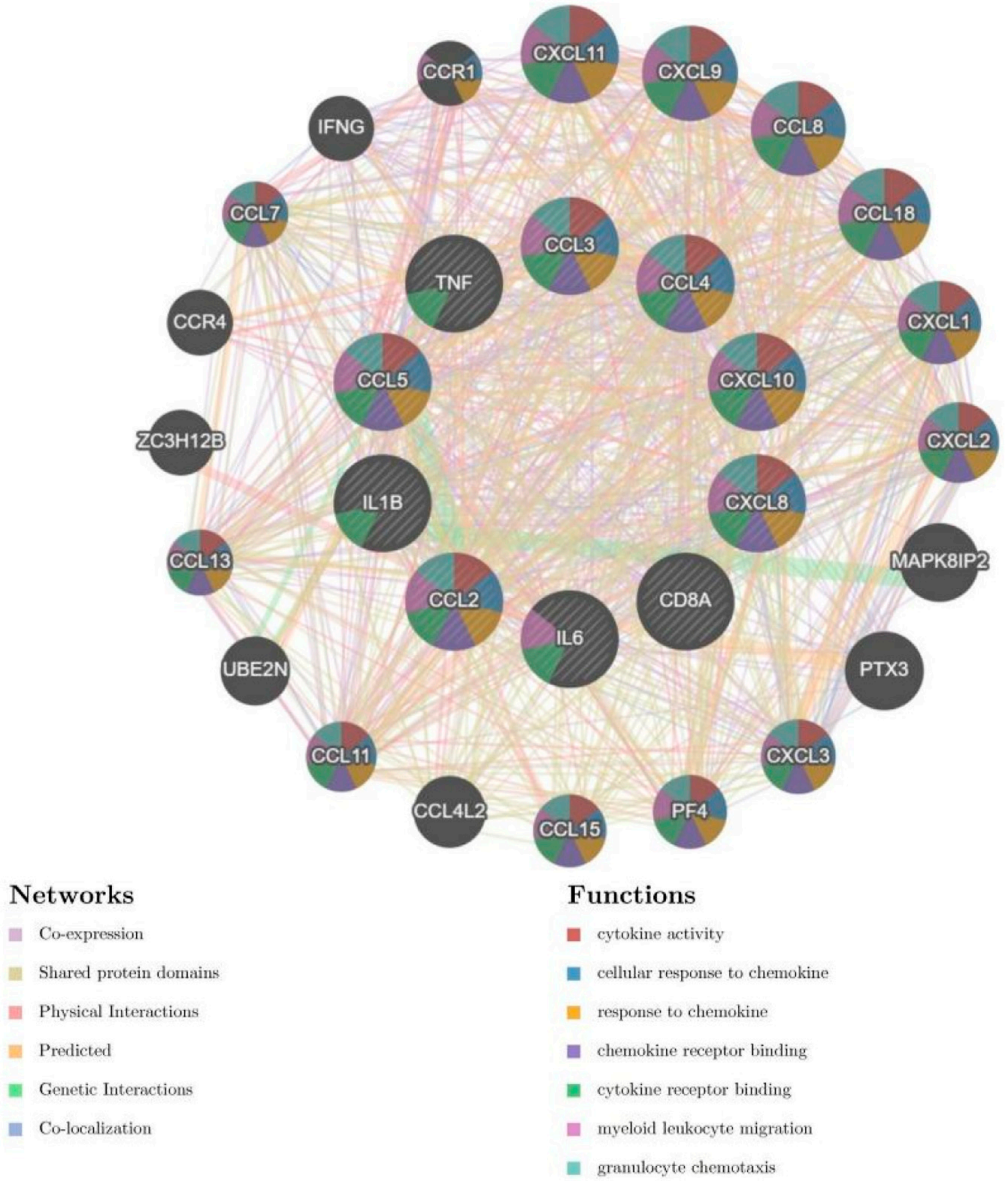
Prediction of gene interactions on hub genes.

The inter-gene prediction of the screened 10 hub genes (Figure 14) showed that 58 genes were interrelated with these 10 hub genes.

### 2.10 Reciprocal prediction between genes and drugs

**FIGURE 15:**
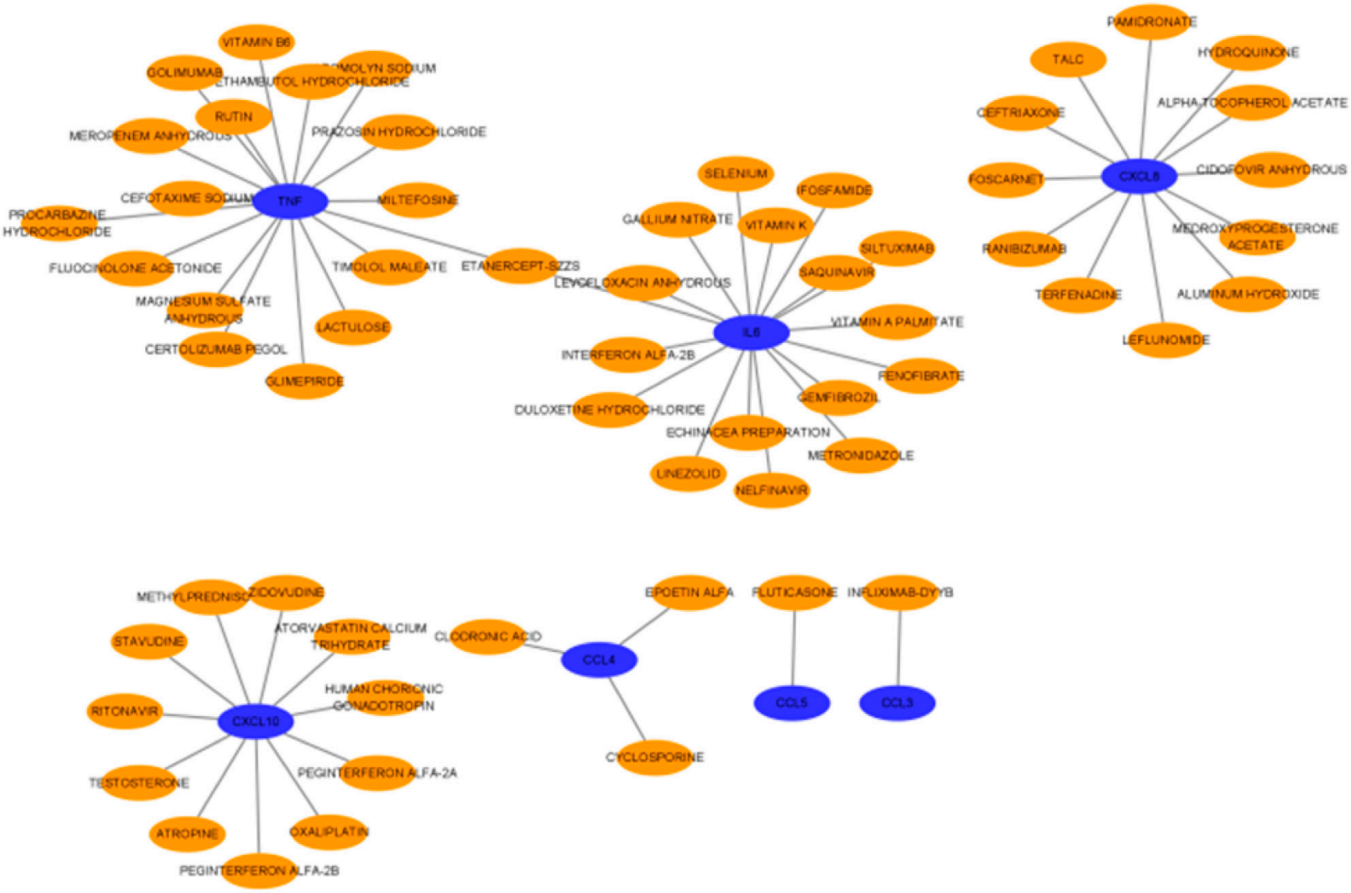
Prediction of drug–gene interactions on hub genes.

After drug–drug interactions were predicted for the screened hub genes (Figure 15), the results showed that 7 genes had interactions with drugs, including a total of 17 medications associated with TNF, 17 drugs acting on IL-6, 12 drugs acting on CXCL8, 11 drugs acting on CXCL10, 3 drugs acting on CCL4, and 1 drug each acting on CCL3 and CCL5. There is one drug that acts on both TNF and IL-6.

### 2.11 miRNA prediction of hub genes

**FIGURE 16:**
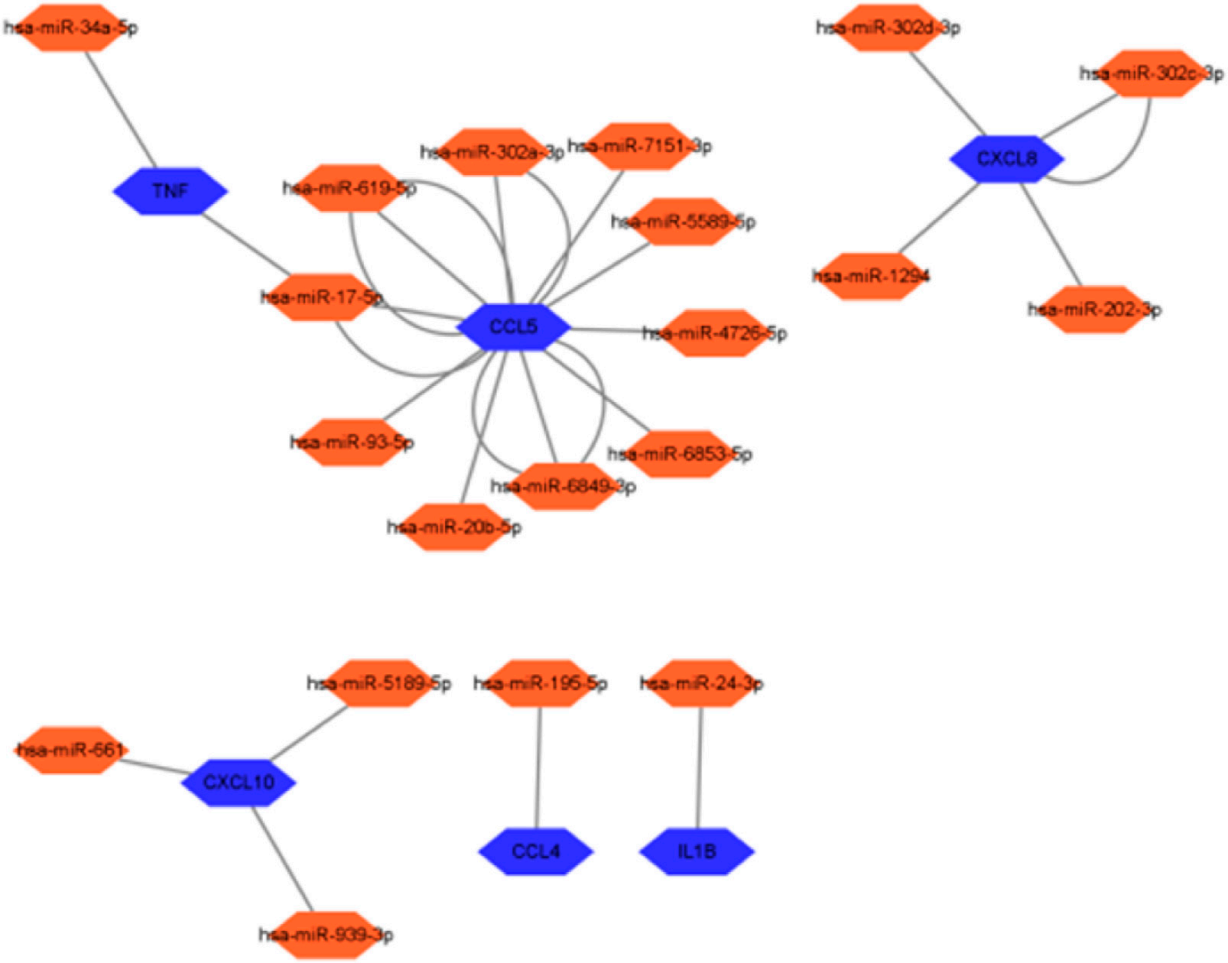
miRNA expression prediction on hub genes.

Further screening of the microRNA (miRNA) prediction results of the 10 hub genes screened (Figure 16) showed that ten miRNAs might be regulated by CCL5, four by CXCL8, three by CXCL10, two by TNF, one by CCL4, and one by IL-1B. IL-1B may be regulated by one miRNA, whereas TNF and CCL5 may share a common miRNA.

### 2.12 Initial validation of the hub gene

**FIGURE 17:**
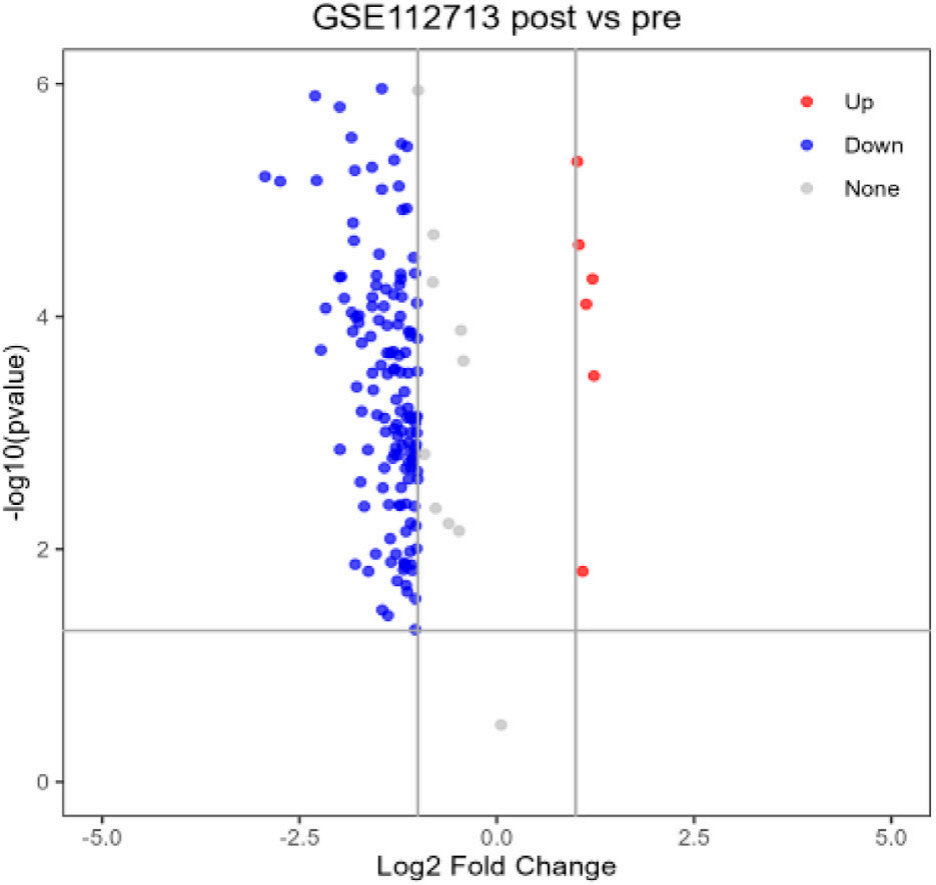
Volcano chart mapped using 166 HIRI-related DEGs.

**FIGURE 18:**
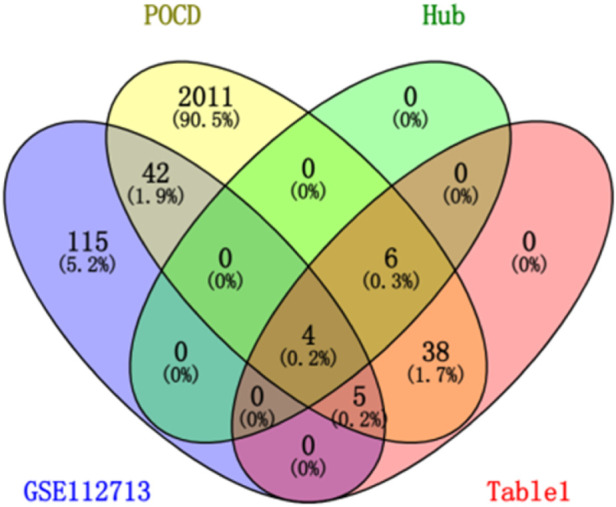
Venn chart of “POCD,” “Table 1” “GSE112713,” and “hub gene.”

After differential analysis and volcano plotting of the dataset GSE112713 (Figure 17), the results showed 166 DEGs, including 161 upregulated genes and 5 downregulated genes. Compared with the 2,053 genes in the POCD-related genes, 51 were initially validated, including *HSPA1A*, *CD83*, *HBEGF*, *KLF5*, and *CALCA*. Comparing with 53 genes in Table 1, 9 of them were preliminarily validated, namely, *TNFAIP3*, *PTGS2*, *LIF*, *BMP2*, *PTX3*, *IL-6*, *CCL2*, *IL1-B*, and *CCL3*. Compared with 10 hub genes screened in Table 2, *IL-6*, *CCL2*, *IL1-B*, and *CCL3* were preliminarily validated (Figure 18).

## 3 Discussion

HIRI has been the focus of several studies in recent years due to its role in various clinical situations. With the implementation of vascular surgical techniques in liver surgery, HIRI is recognized as a key contributor to postoperative morbidity and mortality (Jaeschke, 2003) because it not only causes liver dysfunction but also damages distant organs, especially the brain (Na et al., 2014; Tong et al., 2018; Jochmans et al., 2017).

Previous studies have demonstrated that hepatic ischemia–reperfusion injury can induce damage to distant organs, including the hippocampus and cortex, through activation of NLRP3 inflammatory vesicles and neuronal focal death (Zhang et al., 2019). It has also been suggested that abnormal mitochondrial dynamics may be a potential mechanism for HIRI-induced hippocampal injury and cognitive dysfunction (Yu et al., 2020). However, the specific mechanism of cognitive dysfunction caused by HIRI remains unclear. Therefore, this study theoretically investigated the relationship between HIRI and POCD through bioinformatics analysis.

Our study shows that the expression of relevant gene levels in the liver after a period of ischemia is significantly different before and after reperfusion. We defined the genes that satisfy *p*-value < 0.05 and |logFC| ≥ 1 as DEGs. These DEGs determine the proteins with differential expression. It can be found from the volcano plot in Figure 1 that the vast majority of these DEGs are downregulated genes. So, we believe that the differentially expressed proteins encoded by DEGs may be one of the causes of HIRI. Among these DEGs, 53 were POCD-related genes (Figure 3) (Table 1). Previous studies have found that these 53 genes are involved in the occurrence and development of cognitive dysfunction through multiple mechanisms, mainly including neuroinflammation, immune cell infiltration, blood–brain barrier (BBB) damage, and oxidative stress. Among these POCD-related genes, 3 genes had correlation scores of 20 or more, 6 genes had correlation scores of 10 or more, and 13 genes had correlation scores of 5 or more. Although there were also genes with correlation scores below 5, they provided us with the necessary conditions to further validate POCD and HIRI. In the PPI network constructed for these 53 genes (Figure 8), there was a strong expression linkage between the genes, but the *CYP17A1*, *JPH1*, and *CACNA1H* genes were not linked to the other genes and thus could be used as targets for elimination in subsequent studies. We analyzed the PPI network again using statistical methods (Figure 8), narrowed it down to screen again (Figure 9), and screened out 10 hub genes (Table 2). The GO analysis of the 10 hub genes (Figure 12), along with KEGG analysis (Figure 13), provides an important basis for our future research. In the protein interaction prediction of hub genes, 53 genes interacted were identified, which were co-expressed in some aspects, which provides direction for our subsequent studies. We applied the research data to the dataset GSE112713 for preliminary validation of our study. As shown in Figure 18, the POCD-related genes in this dataset overlap with the 53 genes we screened in Table 1 and contain 4 of the hub genes we screened. The results further suggest that POCD plays an important role in HIRI with these 10 genes.

CCL2, also known as monocyte chemoattractant protein 1 (MCP-1), is a member of the CC subtype chemokine family and acts through its cognate receptor, chemokine receptor 2 (CCR2) (Bose and Cho, 2013). CCL2 expression is elevated in a variety of diseases characterized by acute and chronic inflammation (Cerri et al., 2016). Moreover, HIRI leads to a severe inflammatory response in the body. It has been previously shown that surgically induced upregulation of CCL2 expression in activated astrocytes promotes microglia activation and M1 polarization, increasing pro-inflammatory cytokines and hippocampal neuronal damage (Xu et al., 2017). In HIRI, CCL2 expression shows a significant upregulation and is considered an important DEG (Zhang et al., 2016). In a comprehensive study on gene microarray analysis of expression profiles in HIRI (Zheng et al., 2017), it has been shown that CCL2 is an upregulated DEG. This study reveals that CCL2 may play an important role in HIRI with potential clinical implications. In our research, CCL2 was the hub gene, so we hypothesized that CCL2 plays an important role in HIRI-induced POCD.

Interleukin-1B (IL-1B) is a potent pro-inflammatory cytokine essential for host defense responses against infection and injury (Lopez-Castejon and Brough, 2011). IL-1B is required for the induction of CCL2 (Burke et al., 2012). In a bioinformatics analysis of the pathogenesis of postoperative cognitive dysfunction (Bhuiyan et al., 2022), IL-1B was found to play an important role. In our study, CCL2 and IL-1B were screened as hub genes in the bioinformatics analysis of HIRI leading to POCD; they were intricately related in the predicted PPI network–gene interactions, and when scored using the CytoHubba functional MCC algorithm in Cytoscape software, both CCL2 and IL-1B received equally high scores. Meanwhile, the correlation scores of CCL2 and IL-1B in the GeneCards database were 18 and 27, respectively, ranking 66th and 22nd among all 2,106 genes. Therefore, we can hypothesize that CCL2 and IL-1B play very important roles in POCD caused by HIRI. In addition, a study analyzing potential immune-related genes involved in the pathogenesis of ischemia–reperfusion injury after liver transplantation identified nine genes (Guo et al., 2023), two of which—CCL4 and CXCL8—were also among the hub genes predicted in our study. CXCL8 has a correlation score of 18.8 in the GeneCards database, thus reinforcing that HIRI leads to the development of POCD.

How to mitigate or prevent the occurrence of POCD after HIRI has been the focus of research in clinical work and is also the most important concern of clinical staff. HIRI is one of the causes of POCD, and inhibition of HIRI can alleviate the occurrence of POCD. Some studies have shown (Wu et al., 2019) that rats with a short duration of liver ischemia–reperfusion exhibit less cognitive impairment than those with a longer ischemia duration. However, there are fewer studies on pharmacologic aspects. Therefore, we surveyed the related elements and predicted drug–gene interactions for the 10 hub genes we screened; these predicted drugs can provide a reference for the targeted treatment of POCD in future clinical work.

miRNAs, which are non-coding RNAs approximately 21 nucleotides long, are key post-transcriptional regulators of gene expression in postnatal animals, plants, and protozoa. In mammals, miRNAs are predicted to control the activity of more than 60% of all protein-coding genes (Friedman et al., 2009). It has very powerful physiological roles in fine regulation of gene expression, controlling early development, cell proliferation, apoptosis, cell death, lipid metabolism, and cell differentiation, which directly affect tissues, organs, and even our entire system (Saliminejad et al., 2019). miRNAs play an essential role in regulating the translation and transcription of genes in various ways, such as target mRNA degradation and inhibition of target gene translation (Yazit et al., 2020). miRNAs also play important roles in developing the nervous system, memory, and learning and can potentially cause neurological disorders (Wei et al., 2017). A study has demonstrated that miR-181b-5p can attenuate early POCD by suppressing hippocampal neuroinflammation in mice (Lu et al., 2019). In this study, the prediction of related miRNA expression of 10 hub genes (Figure 16) further revealed the relationship between HIRI and POCD, which can be used to study the specific mechanism by which HIRI leads to POCD and may provide new ideas for mitigating the occurrence of POCD in clinical work. The miRNAs analyzed in this study were compared with those identified in a bioinformatics analysis investigating the role of ferroptosis (iron-dependent cell death) in hepatic ischemia–reperfusion injury (Sun et al., 2022). The miRNAs predicted in this study showed both similarities and differences. For the hsa-miRNA-24-3p that we predicted, there was a study that showed that the use of rosuvastatin alleviated ischemia/reperfusion injury in cardiomyocytes by downregulating hsa-miR-24-3p to target the upregulated uncoupling protein 2 (UCP2) (Wei et al., 2019). Our study predicted has-miRNA-34a-5p, with studies confirming that miR-34a-5p may protect the liver from ischemia/reperfusion injury by inhibiting the JNK/P38 signaling pathway through the downregulation of hepatocyte nuclear factor 4α (HNF4α) (Zheng et al., 2022). Therefore, we hypothesized that regulating miRNA levels may attenuate the occurrence of postoperative cognitive impairment by alleviating HIRI.

Although we have conducted extensive research on the correlation between HIRI and POCD, this study still has some limitations. First, we have only used human genes to hypothesize the relationship between HIRI and POCD and have not studied it in animals. In addition, we analyzed HIRI and POCD using only theory, but the biological functions and roles of related genes need to be further investigated by *in vivo* modeling. Finally, there are some limitations in our data selection. We only selected HIRI in liver transplantation and could not include all the conditions leading to HIRI. Therefore, these situations are the focus of our future research and discussion, and we need to refine the relevant experiments to corroborate the conclusions we have hypothesized. Despite the limitations, our findings provide preliminary clues to investigate the relationship between HIRI and POCD and improve and prevent the occurrence of postoperative cognitive impairment in patients.

## 4 Materials and methods

### 4.1 Data sources

The Gene Expression Omnibus (GEO) database (https://www.ncbi.nlm.nih.gov/geo/) is a public gene expression database created by the National Center for Biotechnology Information (NCBI). A public gene expression database contains high-throughput gene expression data and microarray gene expression data (Edgar et al., 2002). We selected the GSE202565 dataset from this database for bioinformatics analysis of genes related to POCD. The GSE202565 dataset is from a study on the administration of the irreversible pan-cysteine asparaginase inhibitor during ambient machine perfusion of the liver, which attenuates innate immune and pro-inflammatory responses in a non-*in-situ* environment. We chose to include a set of data: eleven livers from a study cohort of human livers discarded after prior machine perfusion served as the control group and five consecutively recruited human livers rejected for transplantation served as the experimental group, which received emricasan at a dose of 5 mg/kg liver weight before the start of hepatic perfusion. Empty core needle biopsies for transcriptome sequencing were performed before perfusion and 3 and 6 h after perfusion (Raigani et al., 2022).

The GeneCards database is a comprehensive and authoritative compendium of annotated information on human genes that, and it has been widely used for nearly 15 years. Its gene-centered content is automatically mined and integrated from over 80 digital sources (www.genecards.org) (Safran et al., 2010). This database is comprehensive, aiming to provide comprehensive information on the human genome. It integrates a large number of data sources worldwide, including genomics, transcriptomics, proteomics, genetics, clinical, and functional information. We downloaded genes related to postoperative cognitive dysfunction from this database and obtained 2,053 POCD-related genes.

### 4.2 Bioinformatics analysis methods

#### 4.2.1 Differential analysis of HIRI-related genes and mapping of volcanoes

We selected data with a reperfusion time of 3 h from the GSE202565 dataset as samples. The selected samples were subjected to differential analysis of gene expression data before and after liver reperfusion using the GEO2R online tool (Barrett et al., 2012) in the GEO database. We plotted a volcano plot with a *p*-value < 0.05 and |logFC| ≥ 1 as a condition.

#### 4.2.2 Acquisition of POCD-related genes and mapping of Venn

We searched the GeneCards database using the search term POCD and obtained 2,053 POCD-related genes; these genes were further analyzed using the VENNY website (version: 2.1) (https://bioinfogp.cnb.csic.es/tools/venny/index.html) (Oliveros, 2022). The obtained DEGs of HIRI and POCD-related genes were taken as intersections and plotted in Venn plots.

#### 4.2.3 GO and KEGG analyses

GO enrichment analysis is a commonly used bioinformatics method for searching large-scale genetic data (including BP, CC, and MF) for comprehensive information. KEGG pathway enrichment analysis is widely used to understand the biological mechanisms and functions of genes/proteins in biological processes (Dong et al., 2021).

We obtained the intersecting genes between the HIRI- and POCD-related DEGs described above using R software (version: 4.3.1) and the following packages: clusterProfiler (version: 4.4.4) (R Core Team, 2022) (Wu et al., 2021), org.Hs.eg.db (version: 3.15.0) (Marc, 2022), enrichplot (version: 1.16.2) (Yu, 2022), and ggplot2 (version: 3.3.6) (Wickham, 2016). The KEGG and GO analyses were performed using a p-value <0.05. The obtained results of GO and KEGG analyses were sorted in order from smallest to largest, and the top 10 results of the selected GO analysis and the top 30 results of KEGG analysis were plotted as bar charts and bubble charts, respectively.

#### 4.2.4 Constructing protein–protein interaction networks

The STRING database (version: 11.5) (https://cn.string-db.org/) is a systematic collection and integration of protein–protein interactions, including physical interactions and functional associations (Szklarczyk et al., 2023). We put the DEGs of HIRI and the intersecting genes obtained from POCD-related genes into this database for PPI network construction.

#### 4.2.5 Cytoscape software to analyze PPI networks and screen hub genes

Cytoscape software (version 3.9.1) graphically displays, analyzes, and edits networks (Shannon et al., 2003). We used the CytoHubba application within Cytoscape to analyze the PPI network and applied the widely used MCC algorithm to screen for hub genes (Chin et al., 2014).

#### 4.2.6 Hub gene GO and KEGG analyses

The screened hub genes were subjected to GO and KEGG analyses again in the same way as described in Section 2.3. Bubble and bar graphs were also obtained.

#### 4.2.7 Prediction of inter-gene interactions

The GeneMANIA database (http://genemania.org/) can help predict gene interactions (Dong et al., 2021). We can use this database to predict interactions between genes related to hub genes.

#### 4.2.8 Prediction of drug–gene interactions

The DGIdb database (version: 4.2.0) (https://www.dgidb.org/) is a publicly accessible resource that compiles information on genes or gene products, drugs, and drug–gene interactions (Cannon et al., 2024). We use this database to predict drugs with which hub genes are associated. The resulting data were screened under the conditions of registration approval status as approved and an interaction score of 10. The screened data were visualized using Cytoscape software (version 3.9.1).

#### 4.2.9 miRNA prediction

The Norwalk database (version 2.0) (http://mirwalk.umm.uni-heidelberg.de/) enables the prediction of interactions between genes and miRNAs (Sticht et al., 2018). We used this database for the correlation prediction of miRNA expression of hub genes, further filtered the results using validated as a filtering condition, and finally visualized them using Cytoscape software (version: 3.9.1).

#### 4.2.10 Initial validation of the hub gene

We selected the dataset GSE112713 from the GEO database for initial validation of the previously screened hub genes. The GSE112723 dataset is from a study on ambient machine perfusion to inhibit pro-inflammatory responses and promote liver regeneration. We selected one of the datasets: liver transplant patients; transplanted liver donors were preserved using the traditional cold storage method, and liver tissues were extracted for microarray gene expression analysis at the end of the preservation before reperfusion and 60 min after reperfusion, with a total of 11 sets of samples (Sun et al., 2022). A total of 11 sample sets were analyzed using GEO2R in the GEO database (Barrett et al., 2012) to identify difference expression; volcano plots were generated using a *p*-value < 0.05 and |logFC| ≥ 1. Finally, the genes related to POCD, the genes in Table 1, and the hub genes were analyzed using the VENNY website (version: 2.1) (https://bioinfogp.cnb.csic.es/tools/venny/index.html) (Oliveros, 2022). Online Venn plots were drawn for preliminary validation of hub genes.

## 5 Conclusion

Liver ischemia–reperfusion is one of the leading causes of liver injury in liver surgery. It can severely impair the cognitive function of patients in the postoperative period. This study shows that 53 DEGs and hub genes associated with HIRI, screened using bioinformatics methods, were closely related to POCD. The bioinformatics method of analyzing these genes helps further reveal the relevant mechanisms of HIRI leading to postoperative cognitive impairment. It provides valuable reference information for future in-depth research by other investigators. The targeted drugs and miRNAs predicted for the 10 hub genes provide reference value for clinical efforts to reduce the incidence of cognitive impairment after liver surgery and lessen the economic burden on patients. The 10 hub genes can be used as therapeutic targets for POCD and become essential resources for future research on the pathogenesis, diagnosis, and therapeutic intervention of POCD.

## Data Availability

The datasets presented in this study can be found in online repositories. The names of the repository/repositories and accession number(s) can be found in the article/supplementary material.
